# Migration as a risk factor for school dropout amongst children made vulnerable by HIV/AIDS: a prospective study in eastern Zimbabwe

**DOI:** 10.1080/17450128.2015.1034799

**Published:** 2015-04-22

**Authors:** Erica L. Pufall, Constance Nyamukapa, Laura Robertson, Paradzai George Mushore, Albert Takaruza, Simon Gregson

**Affiliations:** ^a^ Department of Infectious Disease Epidemiology, Imperial College London, St. Mary’s Campus, Norfolk Place, London, UK, W2 1PG; ^b^ Biomedical Research & Training Institute, No. 10 Seagrave Road, Avondale, Harare, Zimbabwe

**Keywords:** education, migration, HIV, children, orphans, Zimbabwe

## Abstract

Orphans and other children made vulnerable by HIV in sub-Saharan Africa are at increased risk of moving household and of dropping out of school. However, the relationship between child migration and school enrolment has not been established. Multivariable regression models and prospective data from a cohort of children in Manicaland, Zimbabwe, were used to investigate the effect of migration on school enrolment. Children who had moved household were at increased risk of dropping out of school after adjusting for orphan status, relationship to primary caregiver, and household wealth. Interventions are needed to ensure that children who migrate are re-enrolled in school.

## Introduction

The HIV epidemic in sub-Saharan Africa has increased the prevalence of orphanhood and the number of other vulnerable children, as a result of HIV-related illness, stigma, and death within households and communities (Barnett & Blaikie, 1992; UNAIDS, 2010; UNICEF, 2012). Orphans and other vulnerable children (OVC) have been found to be at increased risk of poor educational outcomes (Beegle, De Weerdt, & Dercon, 2009; Bicego, Rutstein, & Johnson, 2003; Case & Ardington, 2006; Case, Paxson, & Ableidinger, 2004; Monasch & Boerma, 2004; Parikh et al., 2007; Timaeus & Boler, 2007). Furthermore, a number of studies have found that lower educational attainment can be associated with higher risk of acquiring HIV infection (Hargreaves et al., 2008). In Zimbabwe, orphan status, particularly maternal orphanhood, has been associated with lower primary school completion rates (Nyamukapa, Foster, & Gregson, 2003; Nyamukapa & Gregson, 2005) and, amongst adolescent girls, lack of secondary school education (Gregson et al., 2005), being out of school, and poorer educational attainment (Birdthistle et al., 2009). However, the mechanisms through which orphanhood and vulnerability affect school enrolment and education outcomes are not well described.

One possibility is that increased migration amongst OVC, due to parental loss and changes in caregiver, might contribute to greater school dropout. Migration may lead to increased school dropout for a number of reasons, including a weakening of peer networks (South, Haynie, & Bose, 2007), lower social and academic engagement (Rumberger & Larson, 1998), schools not accepting new pupils (Batbaatar et al., 2006), and schools requiring documentation that migrants may lack (Porteus et al., 2000). Longer distances of migration may also pose a problem to pupils as they will have lost access to the social capital and community from which they come, and may also need to cope with the change from a rural to urban environment, or vice versa (Goksen & Cemalcilar, 2010; Hunt, 2008). Moreover, larger migration distances may mean that a child moves out of their current school’s catchment area, requiring them to re-enrol in school, a problem that would not occur if they moved within the same village, for example. In sub-Saharan Africa, orphans are usually cared for within the extended family, which is thought to have a number of benefits for children’s well-being and development (Foster & Williamson, 2000). However, recent qualitative work (Ansell & Young, 2004; Young & Ansell, 2003) from southern Africa has highlighted the potential negative impacts of AIDS orphans migrating to live with widely dispersed extended family members, including mistreatment by foster families and interruption of education.

In this paper, we used prospective data, collected between 2002 and 2006, from a stratified cohort of orphaned and non-orphaned children from Manicaland, eastern Zimbabwe, to investigate whether children who recently migrated were at increased risk of dropping out of school. We also investigated associations between orphanhood, type of caregiver, and school dropout and whether any such associations are explained by an increased risk of migration amongst affected children.

## Methods

Since 1998, five rounds of a population-based, open, prospective cohort study (Gregson et al., 2006) have been conducted in 12 study sites in Manicaland province, eastern Zimbabwe. At each round of this survey, a census of all households in the area is conducted. All household members are enumerated and basic demographic information is collected, including the orphan status of all children less than 18 years old living in the household. Adults aged 15–54 years are then invited to complete a detailed Individual Questionnaire, which includes questions on sexual behaviour and HIV risk.

Three rounds of an embedded child cohort study were conducted in eight of the study sites (two agricultural estates, two roadside trading settlements, two small towns, and two subsistence farming areas) between 2002 and 2006. In the second round of the household census (2001/2003), all maternal and double orphans, one in five paternal orphans, 1 in 50 non-orphans, and all children under 18 years with recently deceased biological parents were identified and recruited to the embedded child cohort study. The study employed a closed-cohort design and continued for three rounds: December 2002–March 2004; June 2003–November 2004; and July 2004–March 2006. Data were collected on topics including living arrangements, childcare responsibilities, childcare arrangements, extended family, education, child health and nutrition, and psychosocial distress. Questions on childcare arrangements were addressed to the primary caregiver. The remaining questions were answered by the child with help from the primary caregiver if the child was less than 7 years old. The questionnaires for the baseline and follow-up surveys are included as Supplementary Files 1 and 2, respectively.

At the third round of the child cohort study, extra efforts were made to follow-up children who had migrated, both within and outside the study sites. For this reason, we used baseline data from round 2 and follow-up data from round 3 to investigate the association between migration and school dropout amongst children aged 6–18 years who were enrolled in school at round 2. Therefore, we refer herein to the second round of the embedded child cohort study as the baseline survey and the third round of the cohort survey as the follow-up survey. Children were defined as having migrated between survey rounds if they reported moving to a new homestead between the baseline and follow-up surveys.

Univariable associations between school enrolment and demographic characteristics were assessed at baseline using a chi-square test. Multivariable logistic regression was used to investigate whether children who were enrolled in school at baseline, and who migrated between the baseline and follow-up surveys, were more likely to have dropped out of school at follow-up than children who did not move households between the survey rounds. We measured the associations between orphan status, relationship to primary caregiver (natural mother, natural father, grandparent, other close relative, or other), and household wealth tercile (using a previously developed index (Lopman et al., 2007)) and migration between households during the inter-survey period. Two models were developed: the first ‘crude’ model adjusting for the sex and age of the child and community type at baseline (subsistence farming area, agricultural estate, small town, or roadside trading settlement); the second multivariable model adjusting for the effects of all variables. We also tested for interactions between migration and orphan status and migration and socio-economic status to determine if orphans and the poorest children who migrated were more likely to drop out of school than other children.

Next, we measured the associations between migration, the other study variables, and the risk of dropping out of school between baseline and follow-up. Three models were developed: (i) crude analysis – the effects of each variable adjusting as in the above ‘crude’ model; (ii) adjusted analysis (excluding recent migration) – the effects of variables other than migration adjusting for the effects of all variables except migration; and (iii) adjusted analysis (including recent migration) – the effect of migration after adjusting for all of the other variables.

Finally, we investigated if the effect of migration on school dropout differed by distance migrated. Caregivers were asked where their child had lived prior to coming to stay with them. We compared the effects of migration on school dropout amongst children that were reported to have moved within the local area and children that were reported to have moved a greater distance (e.g. from Harare or another town).

Ethical approval for the child cohort study was obtained from St. Mary’s Research Ethics Committee (Number 04/ Q0403/ 130), Biomedical Research and Training Institute (reference number AP65/05), and Medical Research Council of Zimbabwe (reference number MRCZ/A/990).

## Results

A total of 763 children aged 6–18 years were enumerated at baseline (June 2003–November 2004) and 645 (85%) were followed up (July 2004–March 2006). At baseline, over 90% of children were enrolled in school (Table 1). The majority of the children enumerated were orphans due to the oversampling of orphans in the study design. Primary caregivers were most frequently mothers (26%), grandparents (22%), or other close relatives (24%). Of those cared for by a grandparent, 61% and 36% were cared for by a maternal and paternal grandmother, respectively (data not in table). Those cared for by another close relative were most frequently cared for by a maternal aunt (37%), paternal aunt (27%), or by a brother (14%) or sister (18%). Some children were missing data on orphan status (9%) and relationship to caregiver (18%) at baseline. We coded and analysed this missing data and present results for these children in order to increase statistical power and to assess possible biases resulting from missing data.

**Table 1.** Characteristics of the child cohort at baseline (2003/04) and their relationship to school enrolment; *N* = 763.

Characteristic	Percent	*n*	Percent enrolled in school	*p*-value^†^
**Orphan status**				
Non-orphans	18.0%	137	92.7%	0.06
Maternal orphans	14.7%	112	86.6%	
Paternal orphans	25.2%	192	91.7%	
Double orphans	33.3%	254	89.0%	
Missing	8.9%	68	98.5%	
**Relationship to primary caregiver**				
Natural mother	26.1%	199	96.0%	<0.001
Natural father	5.1%	39	94.9%	
Grandparent	22.3%	170	90.6%	
Other close relative	24.4%	186	90.9%	
Other	4.3%	33	48.5%	
Missing	17.8%	136	92.6%	
**Socio-economic status**				
Least poor	33.3%	254	87.4%	0.02
Poorer	33.5%	255	95.3%	
Poorest	33.2%	253	89.7%	
Missing	0.0%	0	N/A	
**Location**				
Agricultural estates	21.1%	161	88.2%	0.01
Small towns	16.5%	126	84.9%	
Roadside trading settlements	28.1%	214	94.9%	
Subsistence farming areas	34.3%	262	92.0%	
Missing	0.0%	0	N/A	
**Age of child**				
6–8 years	16.0%	122	94.3%	<0.001
9–11 years	25.0%	191	98.4%	
12–14 years	28.1%	214	97.2%	
15–16 years	20.1%	153	86.9%	
17–18 years	10.9%	83	59.0%	
Missing	0.0%	0	N/A	
**Sex of child**				
Male	49.8%	380	92.6%	0.09
Female	50.2%	383	89.0%	
Missing	0.0%	0	N/A	
**School enrolment**				
Currently enrolled in school	90.9%	694	N/A	N/A
Currently not enrolled in school	9.1%	69	N/A	
Missing	0.0%	0	N/A	

Note: **^†^** *p*-value for Chi-square test.

Ninety-two percent (591/645) of children enumerated at baseline and follow-up were enrolled in school at baseline and therefore included in the main analysis. School enrolment at baseline was significantly associated with a child’s relationship to their primary caregiver, their socio-economic status, community type, and age, but not their gender or orphan status (Table 1). Eleven percent (50/467) of enrolled children moved household between the two survey rounds and 9% (56/590) had dropped out of school at follow-up. In the crude analyses, maternal orphans were more likely to have migrated than non-orphans (Table 2). Children with any caregiver other than their natural mother were also at increased risk of migration, particularly those being cared for by their natural father. The poorest children were found to be at lower risk of migration than the least poor children. Female children were significantly more likely to have migrated compared to male children.

**Table 2.** Risk factors for migrating between baseline and follow-up rounds of the child cohort study amongst children aged 6–18 years at follow-up who were enrolled in school at baseline.

			Crude analysis*			Adjusted analysis*		
Risk factor	Percent migrated	*N*	AOR*	95% CI	*p*-value	AOR*	95% CI	*p*-value
**Orphan status**								
Non-orphans	4.8%	105	–	–	–	–	–	–
Maternal orphans	18.9%	53	4.23	1.32–13.59	0.015	1.18	0.28–4.99	0.820
Paternal orphans	11.5%	130	2.27	0.77–6.66	0.137	1.97	0.62–6.29	0.251
Double orphans	11.4%	140	2.25	0.78–6.54	0.135	0.64	0.17–2.41	0.509
Missing	10.3%	39	2.13	0.53–8.53	0.287	0.79	0.16–3.96	0.775
**Relationship to primary caregiver**								
Natural mother	5.7%	159	–	–	–	–	–	–
Natural father	18.2%	22	3.88	1.04–14.48	0.044	3.95	0.70–22.25	0.120
Grandparent	12.5%	88	2.47	0.96–6.31	0.060	3.81	1.17–12.47	0.027
Other close relative	17.5%	103	3.49	1.45–8.41	0.005	4.84	1.47–15.98	0.010
Other	27.3%	11	4.47	0.90–22.35	0.068	4.94	0.80–30.59	0.086
Missing	6.0%	84	0.95	0.30–3.00	0.927	1.11	0.33–3.73	0.862
**Socio-economic status**								
Least poor	17.5%	143	–	–	–	–	–	–
Poorer	9.8%	163	0.54	0.27–1.10	0.091	0.56	0.26–1.21	0.140
Poorest	4.5%	155	0.21	0.08–0.52	0.001	0.23	0.09–0.60	0.003
**Location**								
Agricultural estates	13.3%	90	–	–	–	–	–	–
Small towns	14.5%	76	1.24	0.50–3.07	0.647	0.93	0.34–2.53	0.885
Roadside trading settlements	10.0%	140	0.76	0.33–1.77	0.524	0.69	0.27–1.77	0.444
Subsistence farming areas	8.1%	161	0.61	0.26–1.42	0.247	0.53	0.21–1.33	0.176
**Age of child**								
6–8 years	10.9%	55	–	–	–	–	–	–
9–11 years	8.3%	120	0.73	0.25–2.17	0.574	0.67	0.20–2.25	0.516
12–14 years	8.0%	137	0.74	0.26–2.12	0.571	0.54	0.16–1.80	0.316
15–16 years	11.4%	88	1.00	0.34–2.99	0.992	0.56	0.16–1.99	0.368
17–18 years	20.0%	65	2.14	0.74–6.22	0.162	1.46	0.41–5.18	0.560
**Sex of child**								
Male	8.4%	251	–	–	–	–	–	–
Female	13.4%	216	1.85	1.00–3.42	0.048	2.26	1.16–4.40	0.016

Notes: *The crude logistic regression model adjusts for age and sex of the child at follow-up and location at baseline; the adjusted model adjusts for all variables.

In the full multivariable model, the strength of the associations between all types of orphanhood and migration was reduced and was no longer statistically significant (Table 2). The effect of relationship to primary caregiver increased in the multivariable model, with children being cared for by their grandparents becoming more likely to have migrated between baseline and follow-up. The poorest children continued to be at reduced risk of migrating compared to the least poor children in the adjusted analyses. Female children continued to be more likely to migrate than male children. Children with missing data on orphanhood and relationship to primary caregiver were not significantly at risk of migrating in the multivariable model.

A variety of reasons were given for why children who migrated had come to live in their current household: mother’s or caregiver’s death or illness (21%), adolescent married (8%), child moved to a new school (8%), caregiver recovered after an illness (4%), child mistreated by caregiver (4%), mother moved to new area (2%), left school due to insufficient funds for fees (2%), left school due to failed exams (2%), completed school/passed exams (2%), and other (47%). The majority of children who migrated (74%) moved to households outside their local area.

Table 3 shows the crude and adjusted risk factors for dropping out of school at follow-up amongst children aged 6–18 years who were enrolled in school at baseline. Migration between baseline and follow-up was a strong risk factor for dropping out of school in the crude (AOR, 4.49; 95% CI, 1.94–10.37) and multivariable (AOR, 7.02; 95% CI, 2.76–17.87) models. Orphanhood and relationship to caregiver were not significantly associated with dropping out of school in the crude or adjusted models. Poorer children were found to be at increased risk of dropping out of school, compared to richer children, in the multivariable model, although the poorest children were not at increased risk. Children with missing orphanhood and caregiver data were not found to be at significantly increased risk of dropping out of school. When testing for interactions between migration and either orphanhood or socio-economic status in the full model, we found no significant associations for any of the interaction terms (all *p* > 0.05), and the significance of the variables significant in the full model remained consistent.

**Table 3.** Risk factors for dropping out of school between baseline and follow-up rounds of the child cohort study amongst children aged 6–18 years at follow-up who were enrolled in school at baseline.

Risk factor	Percent dropped out	*N*	Crude analysis*			Adjusted analysis (excluding recent migration)*			Adjusted analysis (including recent migration)*		
			OR	95% CI	*p*-value	OR	95% CI	*p*-value	OR	95% CI	*p*-value
**Migrated between baseline & follow-up?**											
No	7.7%	416	–	–	–	–	–	–	–	–	–
Yes	30.0%	50	4.49	1.94–10.37	<0.001	–	–	–	7.02	2.76–17.87	<0.001
**Orphan status**											
Non-orphans	9.4%	117	–	–	–	–	–	–	–	–	–
Maternal orphans	8.0%	75	0.54	0.17–1.73	0.298	0.67	0.14–3.22	0.619	0.80	0.14–4.53	0.805
Paternal orphans	10.6%	151	0.92	0.36–2.32	0.855	0.90	0.34–2.34	0.822	0.49	0.17–1.42	0.189
Double orphans	9.2%	196	0.62	0.25–1.54	0.307	1.02	0.29–3.59	0.978	0.79	0.18–3.51	0.759
Missing	9.8%	51	0.67	0.19–2.43	0.543	1.21	0.26–5.69	0.814	1.46	0.25–8.56	0.675
**Relationship to primary caregiver**											
Natural mother	11.8%	170	–	–	–	–	–	–	–	–	–
Natural father	10.3%	29	0.87	0.21–3.64	0.846	1.43	0.21–9.85	0.715	0.98	0.11–8.76	0.986
Grandparent	5.2%	136	0.41	0.15–1.09	0.074	0.35	0.09–1.31	0.120	0.38	0.08–1.75	0.213
Other close relative	10.8%	139	0.46	0.20–1.06	0.068	0.49	0.14–1.72	0.265	0.29	0.06–1.35	0.113
Other	33.3%	12	0.61	0.15–2.60	0.508	0.71	0.13–3.82	0.686	0.57	0.08–3.92	0.567
Missing	6.7%	104	0.37	0.14–1.01	0.053	0.42	0.14–1.28	0.126	0.42	0.12–1.52	0.186
**Socio-economic status**											
Least poor	9.7%	185	–	–	–	–	–	–	–	–	–
Poorer	12.4%	202	1.94	0.93–4.02	0.076	1.98	0.90–4.35	0.088	3.33	1.27–8.73	0.014
Poorest	6.6%	196	1.07	0.46–2.47	0.879	1.01	0.42–2.45	0.982	1.70	0.57–5.07	0.338
**Location**											
Agricultural estates	7.1%	113	–	–	–	–	–	–	–	–	–
Small towns	11.0%	91	2.25	0.77–6.59	0.139	2.46	0.79–7.72	0.122	2.02	0.56–7.26	0.281
Roadside trading settlements	11.7%	180	2.15	0.85–5.42	0.106	2.07	0.77–5.51	0.147	2.32	0.79–6.78	0.124
Subsistence farming areas	8.3%	206	1.76	0.68–4.55	0.240	1.72	0.64–4.62	0.282	1.20	0.39–3.72	0.749
**Age of child**											
6–8 years	4.6%	66	–	–	–	–	–	–	–	–	–
9–11 years	0.7%	145	0.14	0.01–1.35	0.088	0.14	0.01–1.38	0.090	0.14	0.01–1.49	0.103
12–14 years	3.8%	183	0.85	0.21–3.39	0.814	0.86	0.22–4.59	0.839	1.02	0.22–4.79	0.981
15–16 years	12.0%	108	2.93	0.79–10.78	0.107	3.17	0.75–13.89	0.097	3.61	0.80–16.21	0.094
17–18 years	37.7%	85	13.1	3.74–46.20	<0.001	15.3	4.47–78.28	<0.001	18.9	4.28–83.29	<0.001
**Sex of child**											
Male	10.0%	310	–	–	–	–	–	–	–	–	–
Female	8.9%	280	1.16	0.62–2.15	0.649	1.05	0.50–2.20	0.904	1.07	0.51–2.26	0.861

Notes: *The crude logistic regression model adjusts for age and sex of the child at follow-up and location at baseline; the adjusted models adjust for all variables, except migration status, which is excluded in the second model, presented here, but is included in the third model.

**Figure 1.**
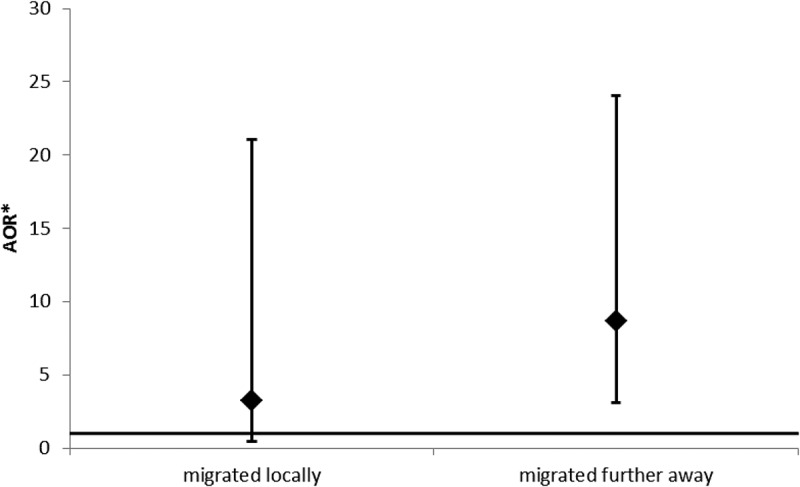
Adjusted odds ratios for dropping out of school comparing children aged 6–18 years who migrated locally, and those who migrated further away, with children who did not migrate between baseline and follow-up.

Figure 1 shows that children who reported migrating ‘locally’ were not significantly more likely to drop out of school compared to children who did not migrate (AOR, 3.22; 95% CI, 0.49–21.08; *p* = 0.2), but children who moved to more distant locations were (OR, 8.64; 95% CI, 3.10–24.08; *p* < 0.001).

*AOR: Adjusted for orphan status, relationship to primary caregiver, wealth tercile and location at baseline and age and sex of child at follow-up.

The most frequent reason for dropping out of school, given by children who did and did not migrate between survey rounds, was insufficient funds – 47% (7/15) and 39% (12/31), respectively. Other reasons given by children who had migrated between study rounds were: marriage (13%), found a job (7%), completed ‘O’ levels (7%), inadequate exam passes (7%), and other (20%). Reasons for dropping out of school given by children who had not migrated between study rounds were: inadequate exam passes (24%), found job (6%), completed ‘O’ levels (6%), illness (6%), left for vocational training (3%), expelled from school (3%), and other (13%). ‘O’ levels are the final year high school exams and five ‘O’ level passes is considered to be a ‘complete’ education, which is the requirement for formal sector employment in Zimbabwe.

## Discussion

In analysing prospective data from a cohort study of OVC in Manicaland, we found that moving household was a significant risk factor for dropping out of school amongst children aged 6–18 years. Furthermore, there was evidence that children who moved larger distances were at the greatest risk of dropping out of school. The prospective, embedded cohort design provides robust evidence for the associations between orphan status, migration, and school enrolment. Many previous studies have relied heavily on cross-sectional data (Bicego et al., 2003; Birdthistle et al., 2009; Gregson et al., 2005; Monasch & Boerma, 2004; Nyamukapa et al., 2003; Nyamukapa & Gregson, 2005), and therefore were not able to fully capture the temporal sequence of events following a demographic change in a child’s life. In our longitudinal dataset, we know that the children who had dropped out of school at follow-up were in school when enumerated previously. However, our study is limited in that we do not know the exact time when a child dropped out of school. It is therefore possible that some dropout events occurred prior to the child moving household. This could compromise our ability to establish a causal relationship between migration and school dropout. However, follow-up time was generally less than a year for most children, so, in most cases, the time between migration and school dropout is likely to have been short. Thus, it is reasonable to infer a causal relationship even if dropout preceded migration. Indeed, we might expect this as families could anticipate a future move and remove children from school some weeks prior to migration. However, it is not possible, in our dataset, to establish with 100% certainty the direction of causation in these cases, although many children reported reasons for migration that were not related to schooling (e.g. death of parents or caregivers). Further longitudinal studies that follow up, over time, migrant children to determine their risks of dropping out of school, and of other adverse outcomes (e.g. sexual risk behaviours) are required.

We had hoped to assess whether more extensive migration amongst OVC contributes to higher levels of school dropout in this group. However, we found only limited evidence in the current study data that orphaned children were more likely to migrate than other children – migration was only statistically significantly more common amongst maternal orphans. In addition, orphaned children were no more likely than other children to have dropped out of school during the follow-up period even before controlling for differences in migration, and migration in orphans was not more likely to be associated with school dropout than in non-orphans. In previous cross-sectional studies in this population, we have found evidence for increased school dropout rates amongst OVC (Nyamukapa et al., 2003; Nyamukapa & Gregson, 2005). This apparent discrepancy may be because the highest risks of school dropout occur shortly before or after the death of a parent (i.e. before enrolment as an orphan in the current study). If so, our finding of a strong relationship between migration and school dropout may still explain, at least to some extent, cross-sectional observations of associations between parental loss and non-enrolment in school.

Given that we found that migration is a strong risk factor for children dropping out of school, it is important to identify ways in which to either decrease child migration and/or mitigate its negative effects on schooling. Interventions that support households caring for OVC could be one possible way to help address this problem. Cash transfer programmes can help overcome financial barriers to school enrolment (e.g. fees, uniforms, and books), and conditioning the cash on school attendance may provide a further incentive for households to enrol OVC in school (Baird, Garfein, McIntosh, & Ozler, 2012; Baird, McIntosh, & Ozler, 2010). Cash transfer programmes, and other structural interventions (e.g. community cash transfers (Skovdal, Mwasiaji, Webale, & Tomkins, 2011) or microfinance programmes (Pronyk et al., 2006)) could also help to prevent child migration in the first place: a regular income might increase household stability and prevent dissolution following economic or demographic shocks. Further research is required to determine whether interventions to stabilize households can reduce school dropouts amongst OVC in sub-Saharan Africa.

The main strength of the study was the follow-up children of who had migrated outside of the study area: we were able to follow up a high proportion of the children we enumerated (85%), which should reduce selection bias due to the generally non-random nature of losses to follow-up. Highly mobile children are often missed in studies where such efforts are not made, usually due to logistical constraints. Furthermore, maternal and double orphans were oversampled in our study, ensuring a large enough sample of these children to make robust statistical inferences regarding their characteristics. These types of orphanhood are relatively rare and demographic surveys are often unable to enumerate large samples of these orphans. For the purposes of making policy and planning interventions for vulnerable children, it is important to have robust information about hard-to-reach, high-risk children.

## Conclusion

It is clear that the time following migration is one of high risk for school dropout amongst children in Zimbabwe – children who moved household were seven times more likely to drop out of school compared to those who did not move household. Interventions are required that target children who have recently migrated to ensure that they can return to school as quickly as possible. It is also important to reduce administrative barriers to school re-enrolment. For example, improving access to birth certificates and other forms of identification may speed up enrolment in school following migration.

## Disclosure statement

All authors have no financial relationships or conflicts of interest relevant to this article to disclose.

## Supplemental data

Supplemental data for this article can be accessed at http://dx.doi.org/10.1080/17450128.2015.1034799
